# The physiology of movement

**DOI:** 10.1186/s40462-020-0192-2

**Published:** 2020-02-04

**Authors:** Steven Goossens, Nicky Wybouw, Thomas Van Leeuwen, Dries Bonte

**Affiliations:** 1 Department of Biology, Ghent University, K.L. Ledeganckstraat 35, 9000 Ghent, Belgium; 2 Laboratory of Agrozoology, Department of Plants and Crops, Ghent University, Coupure Links 653, 9000 Ghent, Belgium

**Keywords:** Body condition, Foraging, Dispersal, Migration, CORT, Hormones, PGI, ATP, Eco-physiological nexus

## Abstract

Movement, from foraging to migration, is known to be under the influence of the environment. The translation of environmental cues to individual movement decision making is determined by an individual’s internal state and anticipated to balance costs and benefits. General body condition, metabolic and hormonal physiology mechanistically underpin this internal state. These physiological determinants are tightly, and often genetically linked with each other and hence central to a mechanistic understanding of movement. We here synthesise the available evidence of the physiological drivers and signatures of movement and review (1) how physiological state as measured in its most coarse way by body condition correlates with movement decisions during foraging, migration and dispersal, (2) how hormonal changes underlie changes in these movement strategies and (3) how these can be linked to molecular pathways.

We reveale that a high body condition facilitates the efficiency of routine foraging, dispersal and migration. Dispersal decision making is, however, in some cases stimulated by a decreased individual condition. Many of the biotic and abiotic stressors that induce movement initiate a physiological cascade in vertebrates through the production of stress hormones. Movement is therefore associated with hormone levels in vertebrates but also insects, often in interaction with factors related to body or social condition. The underlying molecular and physiological mechanisms are currently studied in few model species, and show –in congruence with our insights on the role of body condition- a central role of energy metabolism during glycolysis, and the coupling with timing processes during migration. Molecular insights into the physiological basis of movement remain, however, highly refractory. We finalise this review with a critical reflection on the importance of these physiological feedbacks for a better mechanistic understanding of movement and its effects on ecological dynamics at all levels of biological organization.

## Introduction

An individual-based view on organismal movement as propounded by the Movement Ecology Paradigm (MEP) has provoked a breakthrough in movement ecology as it links the biomechanical and behavioural basis of movement to fitness [1]. The MEP puts three environmentally dependent components of movement forward: motion capacity, navigation capacity, and internal state. As movement operates across different spatiotemporal scales, it can be dissected into its underlying building blocks [2].

**Fig. 1:**
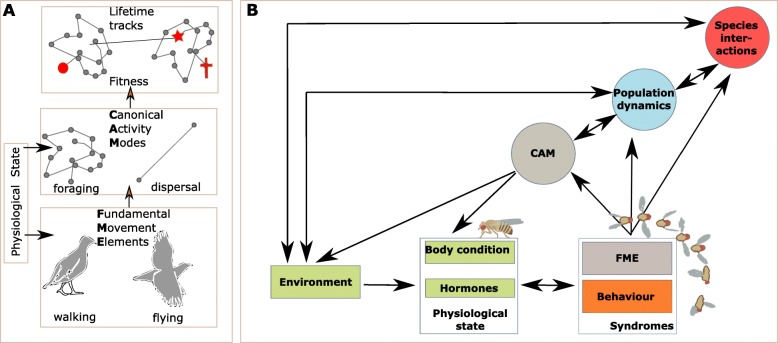
Setting the scene. **a** The physiological state of an individual determines the fundamental elements of movement (FME), as well as an individual’s decision making to switch between different movement modes (CAM) like resting, foraging, dispersing and migrating. Integrated over lifetime, movement is thus central to individual performance, and to fitness across generations. **b** The physiological state of an organism is directly determined by the environment and the elementary (FME) and canonical (CAM) movement modes. Feedbacks among these will affect ecological dynamics at the population and community-level which in turn are anticipated to steer physiology and movement through environmental changes

The Fundamental Movement Elements (FME) form the smallest unit of organismal movement and include for instance step size and wing beat frequency. The FMEs hence depend directly on the motion capacity and internal state components (Fig. 1) and mechanistically integrate into different distinct movement modes [2], referred to as Canonical Activity Modes (CAMs) that are characterized by a distinct movement speed, directionality and correlations of the movement angles. Examples of CAMs include routine foraging, dispersal and migration. Routine movements occur at small temporal and spatial scales with the aim of resource intake, and include displacements at the same scale in response to the same or other species (mate location, predator escape,..). We refer to dispersal as any specific movement during an individual’s lifespan, that make individuals leave the place they were born to a new location where they produce offspring. At short temporal, but usually large spatial scales, individuals can move recurrently between areas in response to environmental cues that predict environmental change. We refer to these movements as migration, and note that despite large distances covered, migration should not result in dispersal as breeding locations may be identical or very closeby among years.

Individuals make decisions to switch between CAMs in response to both the environmental context and internal state. As the sequence and variation in FME’s are strung into an organism’s CAM, any decision made regarding shifts in these CAM’s will depend on an individual’s internal state (and navigation capacity). Because resources are rarely homogeneously distributed in the environment and often continuously changing in time as well, movement will be essential to gain access to resources (such as food, mates and shelter) and will directly impact the individual’s internal state. This feedbacks between an individual’s immediate environment and its internal state will therefore shape its lifetime movement trajectory and fitness [1].

The maximisation of energy balances forms the basis of optimal foraging theory and directly links an individual’s energetic state (body condition) to routine foraging activities [3, 4]. While straightforward from its most fundamental perspective (i.e., the marginal value theorem), we now appreciate that optimal foraging is modulated by environmental factors that have equal or stronger fitness effects, namely predation and disease risk perception and its translation to landscapes of fear and disgust [5, 6]. Foraging movement will thus directly influence energy gain and shape temporal variation in an individual’s internal state. Maximising body condition does, however, not maximise fitness as individuals also have to deal with unpredictable environmental changes at larger spatiotemporal scales. Organisms therefore need to disperse and expose themselves to costs largely exceeding those experienced during routine movements [7, 8]. Movement is thus a fundamental behaviour in life history and the result of a continuous decision making process in terms of how, when and where to displace [1, 9]. Since an individual’s internal state will determine movement, while movement as such will reciprocally affect the individual’s internal state [10], they are tightly connected in a closed feedback loop. Because internal state is closely connected to life histories and behaviour [11], we follow Jachowski and Singh's suggestion to use physiological state as a more accurate term for this internal state [10].

Understanding the causes and consequences of the variation in movement trajectories has been identified as an important knowledge gap in movement ecology [12]. As a first step to integrate feedbacks between movement and physiological state into a formal movement theory, we here provide a view on the current state of the art. More specifically, we synthesise the available evidence on the physiological drivers and signatures of movement. As our aim is to link this condition-dependence to ecology, we do not review the current neurobiological basis of movement decisions as in [13–16], nor the physiology behind wing development in insects [17, 18] but instead provide a synthesis on (1) how physiological state as measured in its most coarse way by body condition correlates with movement decisions related to foraging, migration and dispersal, (2) how changes in stress hormones underlie changes in these movement strategies and (3) whether these can be related to alternative physiological pathways. We finally critically integrate these insights to advance our understanding of the importance of eco-physiological feedbacks in movement ecology and close this review by formulating some unresolved questions.

## Body condition

### From routine movements to dispersal

There is an abundant body of literature on how different movement strategies are related to metrics of body condition. Body condition is predominantly measured in a coarse way by residual or absolute body mass. The efficiency and pace of foraging movements are mostly positively related to a better body condition [19–21]. A good body condition does, however, not necessarily result in longer foraging trips [19]. Rather on the contrary, when foraging costs are substantial, individuals in better body conditions are able to handle prey more efficiently and may show reduced foraging distances [22–24]. Parasites are documented to directly decrease foraging performance by depleting energy reserves and causing physiological damage [25].

Dispersal is a three-stage process, encompassing decision making in terms of departure, displacement and settlement [7, 26]. The social dominance hypothesis predicts emigration of individuals in an inferior physiological state [27]. In house sparrows (*Passer domesticus*), lower ranked individuals leave natal areas earlier than their conspecifics that occupy higher positions in the social hierarchy [28]. Many empirical studies on non-social species report variable relationships between body condition and dispersal [9]. We argue that these different patterns of body condition dependence arise from different levels of spatiotemporal variation of habitat quality. Indeed, theory has shown that costly dispersal is undertaken by individuals in the best body condition in heterogeneous environments where individuals experience variation in fitness prospects [27, 29–31]. This pattern has been widely documented in nature and by means of controlled experiments [32–42]. Interestingly, in metapopulations where local relatedness is high because of low evolved dispersal [27], the opposite Evolutionary Stable Strategy emerges. This has been documented in apterous aphids (*Acyrthosiphon pisum*), where individuals with a decreased energy content dispersed earlier than their siblings in better condition [43].

The eventual dispersal distance and speed is positively associated with a better body condition in insects [44, 45], salamanders [46], fish [47], birds [48–50] and mammals [36]. In two group-living bird species, however, individuals in the best condition remained closest to their place of birth [51, 52]. In a saproxylic beetle (*Osmoderma eremita*) species, flight speed and take-off completion were negatively condition dependent [53]. The unexpected associations are explained by increased advantages of philopatry as familiarity and, hence, fitness prospects in terms of mate finding decrease with distance from the natal range. In a study using money spiders (*Erigone atra*) as a model, emigration has been demonstrated to be positively body condition dependent, with settlement improving under competition in those phenotypes that previously engaged in dispersal [54]. Similar strategies were found in meerkats (*Suricata suricatta*), where individuals in better conditions were found to engage more in prospecting and thereby increased settlement probability [55].

### Migrations and stop-over events

Migration is, like dispersal, a decision making process. If only a certain fraction of the individuals engage in migration, while others remain resident, the strategy is referred to as partial migration. Here, body condition is expected to vary within and among populations and to steer variation in migratory tendency [8]. The three main hypotheses that have been put forward on how body condition may modulate the decision to migrate, are (i) The arrival time hypothesis stating that a migration decision is made when residents have fitness gains by prioritising territory establishment, whereas (ii) the dominance hypothesis states that individuals migrate to escape competition by dominant conspecifics, and lastly (iii), the body-size hypothesis states that a high body condition reduces costs during migration [56]. Both the arrival time and dominance hypothesis predict subordinate individuals to engage in migration, and was found in trout (*Salmo trutta*) [57]. However, other studies focusing on a fish (*Rutilis rutilis*), bird (*Otus elegans botelensis*) and a large mammal herbivore (*Odocoileus hemionus*), did not find an association between migration and within-population heterogeneity in body condition [58–60]. An excellent overview of these hypotheses may be found in Chapman et al. [56] and we refer to Hegemann et al. [58] for a more physiological perspective on partial migration.

Not surprisingly, a body of literature shows that migration trajectories are strongly impacted by the individual’s energetic state. Our insights so far are primarily dominated by research on birds and to a lesser degree migratory fish. Migratory trajectories comprise distances that are magnitudes beyond the daily routine movements and are typically segmented in several migratory movement episodes and stopovers where individuals engage in foraging for refuelling. As energy demands are high to cross these long distances, time spent for stopover activities is higher for individuals in lower body condition [59–71], and hence leads to increased foraging of to allow refuelling [59, 65]. Integrated over the entire migration trajectory, individuals that start migration in better body condition will therefore migrate faster, more directionally and arrive earlier at breeding sites [72–76]. In two anadromous fish species, migration is also negatively related to body condition, [77, 78], but here this correlation is determined by local adaptation to freshwater and hence the upstream breeding grounds.

### A threshold-view on movement decision making

As outlined above, a positive correlation between body condition and the efficiency of routine movements, dispersal and migration has been mostly documented. Efficient movements, do not always translate into longer and faster movements, but instead, evidence is pointing at cost-reducing strategies being the rule for individuals in a good body condition (e.g., [22–24]). Individuals in poor body condition are therefore anticipated to either invest their energy in extended movements or to follow energy-saving strategies by reducing further energy expenditure. Movement-decision making can thus be considered as a threshold trait [79, 80] with individual shifting CAMs when body condition is reaching a specific value. Individuals may adopt in this respect more endurance (thresholds to engage in costly movements at relative high body condition), or conservative (thresholds at low body condition) strategies. Under frequency dependence, both strategies may stably coexist in single populations. While theoretically established [81], it remains to be studied whether such a within-population heterogeneity in movement decision making is effectively related to different strategies adopted in response to body conditions, and whether such fitness stabilising strategies eventually affect population dynamics. Additionally, it remains unclear to which degree physiological constraints overrule this decision-making. Individuals in poor condition might be energetically so depleted that any engagement in extended and beneficial movements might simply not be possible. In kangaroo rats (*Dipodomys spectabilis*), for instance, the timing of emigration is strongly body condition dependent, and only initiated when male individuals reach a critical mass [82]. Feedbacks between movement as both an energy-consuming and an energy-gaining process are thus likely key to spatial behaviours in the wild, but to date poorly understood despite the increase of biologging studies across a wide variety of taxa [83]. Moreover, most insights on such conditional-dependent strategies come from studies that focussed on the active departure phases and neglected decision making in terms of settlement [84]. Given the link between body-condition and competitive ability, it remains to be studied to which degree presumed maladaptive departure decisions may eventually be compensated by facilitated settlement in new environments – especially when demographic and environmental conditions are strongly different between locations.

## Hormones

Body-condition dependent strategies are often overruled by hormonal changes in response to acute biotic and abiotic stressors [85]. We here review the current state-of-the art in order to facilitate the integration of these endogenous processes within a mechanistic movement ecology [70].

### Glucocorticoids in vertebrates

In vertebrates, external triggers of movement decisions such as food shortage, fear and antagonistic interactions with conspecifics are known to initiate a physiological cascade through the hypothalamic-pituitary-adrenal (HPA) axis by which stress hormones (glucocorticoids; abbreviated here as CORT) are released from the adrenal cortex. Creel et al. [85] provide an extensive review on the environmental triggers of this HPA axis activity in social and territorial species. As the main environmental cues of CORT production are known to trigger movement, especially dispersal, it is not surprising that movement is strongly associated with CORT levels, often in interaction with factors related to body or social condition [85].

Food shortage and social interactions attenuate foraging activity through hormonal regulation in birds [86–88]. Elevated CORT levels will equally determine the timing of dispersal in birds and reptiles [86, 88–90]. These elevated hormone levels can be maternally determined [90, 91] and the duration of exposure to maternal CORT amplitudes determines whether individuals stay or disperse [92]. In social vertebrates, increased CORT levels are associated with elevated extra territorial forays, hence with prospecting prior to pre- and dispersal behaviour [93, 94] or with settlement [95].

Baseline plasma CORT levels are elevated in migrating birds to facilitate migratory fattening while protecting skeletal muscle from catabolism, but they also induce health costs [96–100]. The migration modulation hypothesis is brought forward as an explanation of their repressed levels in relation to acute stress during long-distance migration [101]. Studies on partial migration however do not confirm this general pattern [102]. Instead, CORT levels are found to be elevated during landing [101], and increase during stop-over events, where it is positively correlated with fuel loading and behavioural restlessness when active migration is resumed [103, 104]. In nightingales (*Luscinia luscinia*), elevated CORT-levels are modulated by geomagnetic information [105]; and in The European robin (*Erithacus rubecula*) CORT-levels differ between spring and autumn migration [106]. CORT-levels are thus to a large degree externally induced. In dark-eyed juncos (*Junco hyemalis*), genetic variation in these responses was found among two populations overwintering in areas that varied in the level of environmental predictability [107]. More specifically, birds wintering in less predictable and more extreme environments showed a higher amplitude corticosterone response, which may enable them to adjust their behaviour and physiology more rapidly in response to environmental stressors such as storms [107]. Although most studies have targeted CORT, other hormones like ghrelin and melatonin are also known to influence food intake and lipid storage dependent on body condition in migrating birds and other vertebrates [108–110].

### Hormones in insects

Octopamine and adipokinetic hormones are known to regulate energy supply, the oxidative capacity of the flight muscles, heart rate, and probably also a general stimulation of the insect nervous system during periods of intense flight [111]. Octopamine can be considered as the insect counterpart of adrenaline [112]. Although no insect equivalents of corticosteroids have been identified it seems that the adipokinetic hormones perform similar functions [111]. In invertebrates and insects in particular, Juvenile hormone (JH) regulates development, reproduction, diapause, polyphenism, and behaviour [113]. While JH production has been predominantly associated with wing development [114] it has also been shown that lower JH titers advance and increase the duration of flight in corn rootworms (*Diabrotica virgifera*) [115] and milkweed bugs (*Oncopeltus fasciatus*) [116]. In migrant Monarch butterflies (*Danaus plexippus*), migration necessitates the persistence through a long winter season. This prolonged survival has been shown to result from suppressed JH synthesis [117].

## The molecular and physiological basis underlying body condition dependent movement

As outlined above, the dependency of movement strategies on body condition is highly complex and multidimensional, rendering the characterization of the underlying molecular and physiological mechanisms highly refractory. Traditionally, the contribution of candidate genes to foraging, dispersal, and migration behavior has been studied in isolation. We briefly discuss genes of major effect on different movement strategies and subsequently attempt to unify the molecular drivers of movement.

### The usual suspects: genes that greatly influence animal movement

Phosphoglucose isomerase (PGI) is an important metabolic enzyme that catalyzes the reversible second step within the glycolytic pathway. In a series of pioneering studies, Watt and colleagues discovered that different allozymes (different alleles, separable by electrophoresis) of PGI have different thermostabilities in *Colias* butterflies and that their frequencies change in response to heat stress [118–122]. Polymorphisms in the *pgi* gene have subsequently been detected in many insect populations and species [123–125]. Its close association with flight performance rendered *pgi* the ideal candidate gene to study the genetic underpinnings of dispersal ability [123, 126, 127], as for instance in the Glanville fritillary (*Melitaea cinxia*) metapopulation on the Åland island group [128–131]. Currently, a body of work (see review in [132]) identifies PGI and other central metabolic enzymes as prime targets of natural selection via traits related to metabolic rate but also the ability of these enzymes to act as signaling molecules. Collectively, this strongly indicates that a diverse set of central metabolic enzymes determine body condition dependent movement [132].

The central role of a cGMP-activated protein kinase (PKG) in foraging behavior, adult dispersal and perception of nutrient stress in a wide diversity of insect species was initially discovered in the fruit fly *Drosophila melanogaster* where differences in food searching behavior of larvae were mapped to a locus on chromosome-2 called the foraging (*for*) gene [133–139]. It explains the genetic coupling between foraging and conditional dispersal. Since its discovery, homologs of the *for* gene have been studied as a potential causal factor in behavioral transitions in the nematode *Caenorhabditis elegans*, honeybee *Apis mellifera*, and two ant species [140–143]. For instance, upon manipulating the expression of *Amfor* and *egl-f*, orthologs in honeybees and *C. elegans*, respectively, food dependent movement is significantly altered in both species [140, 144].

Clock genes are involved in the timing and onset of migration in birds, fish and butterflies [145–147]. Allelic differences in clock genes like *OtsClock1b* and *Adcyap1* are not only associated with differences in timing and distance of migration but also affect morphology, hormone production and timing of reproduction [146, 148, 149]. Recent work showed that migratory and non-migratory butterflies (*Danaus plexippus*) differ in the Collagen IV alpha-1 gene, which participates in muscle development, metabolism and circadian rhythm pathways [150, 151]. This indicates that a limited number of genes regulate multidimensional traits associated with condition-dependent migration.

### The transcriptomic signature of movement

Although these candidate genes seem to be key regulators for movement behavior, they fail to provide us with a complete insight into the often complex genetic architecture of common traits underlying movement. To overcome this limitation, more pathway-oriented and genome-wide methodologies are now being applied in movement ecology. Advances in –omics technologies not only provide biologists with knowledge concerning the genome-wide gene content of many non-model species, but also the unbiased quantification of transcription by transcriptomics.

Using a transcriptomic approach, Somervuo et al. [152] found a large difference in gene expression profiles between populations of the Glanville fritillary (*Melitaea cinxia*) that inhabit either fragmented or continuous landscapes. These different expression profiles may indicate selection for certain variants in genetic pathways that are involved in successful dispersal in fragmented landscapes [152]. Notably, they found a strong up-regulation in the immune response and down-regulation in the hypoxia response in more dispersive butterflies. The authors attributed this latter transcriptonal shift in dispersive butterflies to a lower sensitivity to changes in oxygen levels, allowing for higher peak metabolic performance during flight before the hypoxia response sets in [152]. Other transcriptomic studies on lepidopterans show similar adaptations to long distance flight on a physiological level, including mobilization of energy, coping with stress (hypoxia) and hormonal control [153]. Transcriptome analysis on adult *D. melanogaster* showed that the *for* gene at least partially operates through the insulin/Tor signaling pathways, which are regulatory pathways that control animal growth, metabolism, and differentiation [137, 154]. In line with the different movement strategies, individual *D. melanogaster* larvae with a long movement path (called rovers) store energy reserves mainly as lipids while individuals with shorter movement paths (sitters) store energy as carbohydrates [136, 137]. In other dipterans with variation in their flight capacity, differential gene expression analysis revealed that the insulin signaling pathway, lipid metabolism, and JH signaling regulate energy during flight [155]. While JH-mediated signaling appears to be an important regulator for migratory behavior in Monarch butterflies (*Danaus plexippus*), no differential expression of the *for* gene was observed [147].

In birds and mammals, transcriptomics offers a new approach to study migration and dispersal by extracting blood from individuals before and after the movement type of interest and comparing RNA profiles. Although this analysis likely excludes important signals from other organs such as the liver and brain, it can offer key insights into molecular mechanisms related to the behavioral decision making of movement. In blackbirds (*Turdus merula*) it was shown that, prior to departure, many genes rapidly change their transcription and these genes are predicted to participate in cholesterol transport and lipid metabolism [156]. In marmots (*Marmota flaviventris*), transcriptomic data shows that the differences between dispersers and resident individuals lie in the upregulation of the metabolism and immunity [157].

### Using metabolomics and gene-editing to find and validate key regulators of movement

Transcriptomic analyses hold great promise to find common underlying molecular pathways that relate to certain types of movement behaviors, but it remains difficult to connect different transcriptomic profiles to the exact levels of metabolite production [158]. In plant-feeding spider mites (*Tetranychus urticae*) that show genetic variation in dispersal along a latitudinal gradient, metabolomic profiling indicated that an allocation of energy could be linked to a dispersal-foraging trade-off, with more dispersive mites evolving to cope with lower essential amino acid concentrations thereby allowing them to survive with lower amounts of food [159, 160]. This finding is consistent with the theory that individuals of a population that forage on the same resources can differ on the genetic level in how these resources are metabolized and that these differences influence their movement behavior [137]. In *Drosophila* that were artificially selected for increased dispersal, higher amounts of octopamine and serotonin were detected [161]. These neurotransmitters are associated with an elevated exploratory behavior in animals, while octopamine is also known to be important when energy reserves have to be mobilized [162, 163]. Octapamine regulates the activation of catabolic enzymes, such as lipases and is the functional equivalent of mammalian norepinephrine [163–165].

No individual genes or single pathway clearly stand out from these metabolomics and transcriptomics studies. To causally link genes to movement, novel gene-editing techniques such as CRISPR/Cas9 technology has now made it possible to modify specific loci within the genomes of many organisms in a stable manner [166]. Gene-editing is not commonly used in ecological research because methodologies are currently time-consuming and highly impractical, especially for complex traits such as movement behavior [167]. Recently, pioneering work of Markert et al. [168] succeeded to efficiently generate and screen heritable clock gene knockout lines in monarch butterflies (*Danaus plexippus*) and recorded changes in migration behavior. Future work needs to incorporate similar gene-editing approaches to advance our understanding of the genetic architecture underlying movement behavior [168].

## Closing the loop

Environmental change imposes physiological changes, but as these determine movement and hence suceptability to these environmental stressors, emerging feedbacks are expected at different levels of biological organisation. First, our synthesis made clear that carry-over effects between the movement modes (CAMs) are very likely. Environmental conditions constraining local foraging will eventually impose physiological changes that limit the efficiency of dispersal and migration events, and reciprocally, any excessive energy expenditure or exposure to additional stressors (if translated into endocrinal reactions) during these long-distance journeys can carry over to foraging movements in the subsequent resident stages [169].

As these physiological changes are anticipated to be correlated with demographic traits and behaviours, hence forming behavioural syndromes [26, 81, 170] they can eventually impact equilibrium population sizes and their fluctuations [171], as mediated by costs during movement and changes in local growth rates (e.g., [172, 173]). Such feedbacks can even be lagged if physiological responses are mediated through maternal effects, as for instance the case by induced hormonal effects [33]. Ultimately, the physiological capacity will determine population dynamic consequences associated with climate change and the persistence of species in an altered environment [91], as for instance demonstrated in the Glanville fritillary [174, 175]. Here, feedbacks between colonisation, extinction and the PGI-related dispersal phenotypes maintained (genetically based) physiological heterogeneity in a metapopulation but since the different genotypes perform differently under different temperatures, gene-flow and metapopulation viability were shown to be vulnerable under climate change [176, 177]. In dendritic systems, body-condition dependent dispersal of a salamander (*Gyrinophilus porphyriticus*) was found to maintain positive growth in putative sinks, hence, contributing to form of self-organisation in these linear habitats [178].

The impact of body-condition dependent movement on community structure has been mainly studied from a co-dispersal perspective, i.e., when hosts in a specific physiological state are moving symbionts. The best-documented consequences of such physiological-induced individual differences are related to the quantity and quality of endozoochorously dispersed seeds by vertebrates [179]. At the other extreme, parasitic symbionts are able to directly modify their host’s physiological state [180] in such a way to manipulate their own spread. Gut bacteria have in this respect been found to steer elementary cell-physiological and hormonal processes along the gut-brain axis that directly modify animal behaviours [181]. Such behavioural modifications are, however, not restricted to gut microbiomes. Presumed commensal *Ricketsia* endosymbionts are for instance found to constrain spider dispersal behaviour [182], while the dispersal limitation in Borellia-infected ticks has been linked to physiological changes that eventually facilitate host transmission of their Lyme-causing bacterial symbiont [183].

## A critical end-reflection

Our understanding of the relative importance of movement-physiology feedbacks in population and community dynamics is still developing. It is nevertheless clear from our review that human-induced rapid environmental changes will affect this eco-physiological nexus, and that the integration of multiple theoretical frameworks may be required to explain the observed variation in movements in nature [184]. Understanding and predicting the responses of animals to environmental change and the potential for solving diverse conservation problems using physiological knowledge is key to the field of conservation physiology [185]. While an extended discussion and speculation on how different anthropogenic pressures affect movement by directly impacting physiological processes is beyond the scope of this review (but see [186] for an excellent contribution with focus on vertebrate migration), we see direct links between spatiotemporal changes in resource quantity and quality, diseases and microbiomes, pollution, invasive species and habitat fragmentation affecting all movement strategies by their impact on body conditions and physiological state. The development of accurate forecasting models is one of the most urgent tasks to guide the effective conservation of biodiversity in the light of global changes. To date, however, models do not provide sufficiently accurate predictions because of an inherent lack of key biological processes, such as physiology and dispersal. We here show that the movement-physiological nexus is such a neglected important mechanism because the direct feedbacks impact connectivity and hence the persistence of metapopulation [187] and the potential for invasions [188].

Unbiased genome-wide transcriptomics using RNA-seq has become very popular in the last couple of years to study movement phenomena [147, 152, 157, 189–191]. In the near future, Next Generation Sequencing (NGS) will allow advanced comparisons of differentially expressed genes across species, movement type, and conditions [192–194]. It is in this respect not unlikely that a new generation of molecular techniques will eventually put the classical classification of dispersal, foraging, and migration aside, while providing a more condition and energy-dependent classification of movement with possible generic molecular responses that unify many types of movement. With the rise of novel molecular tools that allow gene editing [167, 195] and those allowing non-invasive monitoring in wild populations [196], it can also be anticipated that the physiology of movement will be studied at an unprecedented level of detail, especially given the central role of all movement types in species conservation [185].

We obviously applaud this direction as it will advance our understanding of spatial population dynamics substantially from an individual, mechanistic perspective. However, an open question, remains to what extent such highly detailed studies of the physiology of movement are needed or desirable to transform the field of movement ecology towards a more predictive science. It is clear from our review that physiological control mechanisms constrain and dictate variation in how animals with different movement strategies respond to its surrounding environment. The physiological control of movement should therefore be treated as a reaction norm, and as for models including feedbacks between evolution and ecology [197], we expect a realistic but simplified consideration of feedbacks between environmental cues, resources and physiological processes to improve the predictive power of the available models. The integration of simple allometric and metabolic rules offers in this respect promising avenues [171, 198–201], as do dynamic energy budget models [202–204]. It is less obvious to which degree any hormonal feedbacks need to be integrated. There is some evidence that endocrine processes impact direct costs of movement which will potentially impact connectivity at rates that cannot be predicted from metabolic processes alone. Independent of the empirical progress made in understanding the physiological coupling of movement and environmental change, theory is only marginally following this direction. We argue that such a parallel theoretical development is constrained by the added level of complexity, but to date, this has not even been put on the research agenda. Since the few available theoretical studies demonstrated that even the addition of simple movement reaction norms or metabolic rules, can largely change the emerging ecological dynamics, we advocate that a more mechanistic based movement theory is needed more than ever in light of generating synthesis in species responses to global change.

Whether such a theory needs to extend into the molecular pathways underlying the physiology of movement, is more questionable. While this perspective has been recently brought forward within the framework of a predictive ecology in response to climate change [205], our review showed that the needed insights into the principal physiological and genetic drivers of movement are largely lacking. Hence, no theory can be developed without an advanced empirical research agenda.

Modelling approaches that explicitly account for metabolic costs associated with movement and costs associated with risk-taking might already provide general insights on how feedbacks between the environment and physiology eventually shape movement strategies and their coexistence across and within populations (see e.g .[171, 206, 207]). One key area where further insights would benefit ecological forecasting is the study of the putative key- hormones and -genes that are central to the ecophysiological molecular network. If detected, such hormones or genes may serve as master-traits in predictive modelling and improve the accuracy and robustness of mechanistic models by restricting the number of free parameters. We additionally propose theory to integrate movement at lifetime scales, and to focus primarily on behavioural switches between routine movements, dispersal and migration in response to local demographic conditions, body condition and general physiological states (see e.g .[208]) as linkers between local and regional demography. New generations of statistical tools now allow the detection of such discontinuities in movement trajectories [209] and therefore open avenues to use inverse modelling approaches [210] to test the relevance and importance of detailed physiological feedbacks for large-scale individual movement patterns and their impact on population-level processes in a wide array of animals in nature.

## Conclusion

Environments are spatiotemporally heterogeneous, either because of external abiotic drivers or because of internal biotic dynamics. As organisms need to maximise fitness, their movement behaviour should be optimised. Failing to do so might lead to physiological states that constrain such adaptive shifts. Our review demonstrates the central importance of body condition or energetic state as a driver of movements at different spatiotemporal scales, from foraging to dispersal and migration. Overall, as body condition is determined by carry-over effects from early life, we show the importance of these early conditions for physiology and subsequent movement decision making. Negative relationships between movement and body condition become more common with increasing costs of movement. As a decision-making process, the onset of movement at these different spatiotemporal scales is associated with hormonal and gene-expression changes as well. These insights are merely derived from classical model systems and allow a profound insight into the physiological pathways, and the putative correlated responses on other traits and performance. It is, however, clear that much more work is needed to achieve sufficient progress in the field to develop a unifying synthesis on the link between environmental change, physiology and the resulting feedbacks on ecological dynamics. We encourage endeavours in this direction and are hopeful because of the accelerating rate at which new methodologies are developed. However, given the infancy of a physiological movement ecology and the urgency to develop a predictive model of biodiversity in response to environmental change, we advocate a cost-based modelling approach that considers movement decision thresholds in relation to basic physiological states as an important step forward. Ideally, such modelling approaches are centred on physiological dynamics caused by key-molecular pathways, that link environmental change to the condition-dependency of movement across the relevant spatiotemporal scales.

## Synthesis of the outstanding questions

Knowledge caveats hinder the development of a movement ecology that integrates detailed physiological feedbacks in terms of the underlying molecular networks. It remains to be investigated whether and how much the integration of first principles that underlie physiological changes of movement, as to be developed by the next generation of theory, improve the predictive power of ecological forecasting models. Here we summarise the outstanding questions related to the main topics that are discussed in this paper.

1. Questions related to movement-decisions that depend on body condition.
  - How variable are body-condition dependent thresholds across contexts and environments, and to which degree do they underlie heterogeneity in movement strategies within and across populations?
  - What is the impact of these threshold responses on population dynamics and vice-versa?
  - When are these body-condition dependent movements overruled by hormonal processes (e.g., related to predation pressure, fear, social status and other stressors that are mechanistically decoupled from energetic condition)?
  - As metabolic processes and movement allometrically scale to body size, are condition-dependent strategies variable among species of different size, or even trophic levels (e.g. see [205])?
2. Questions related to movement syndromes
  - How are physiological processes that are central in life history and behavior at the basis of movement syndromes, i.e. how do movement strategies correlate with life histories and other behaviour?
  - How are these correlations shaped by the inter- and intraspecific interactions?
  - To what extent can microbial symbionts influence and shape these correlations and movement strategies?
3. Questions related to genes underlying movement-decisions
  - Are there generic molecular pathways that underly many different movement strategies and are they regulated by the same genes and hormones in different species?
  - Is there a common genetic background for movement syndromes, and strategies across all life stages?

## Data Availability

All references are listed in the paper

## Abbreviations

CAM: Canonical Activity Modes
CORT: Glucocorticoids
CoT: Costs of transport
FME: Fundamental Movement Elements
HPA: Hypothalamic-pituitary-adrenal
JH: Juvenile hormone
MEP: Movement Ecology Paradigm
PDF: Neuropeptide pigment dispersing factor
PGI: Phosphoglucose isomerase
PKG: Protein kinase
SNP: Single Nucleotide Polymorphism
